# Interaction of leisure‐time physical activity with body mass index on the risk of obesity‐related cancers: A pooled study

**DOI:** 10.1002/ijc.34011

**Published:** 2022-04-07

**Authors:** Ming Sun, Tone Bjørge, Stanley Teleka, Anders Engeland, Patrik Wennberg, Christel Häggström, Tanja Stocks

**Affiliations:** ^1^ Department of Clinical Sciences in Lund Lund University Lund Sweden; ^2^ Department of Global Public Health and Primary Care University of Bergen Bergen Norway; ^3^ Cancer Registry of Norway Oslo Norway; ^4^ Department of Surgical Sciences Uppsala University Uppsala Sweden; ^5^ Division of Mental and Physical Health Norwegian Institute of Public Health Bergen Norway; ^6^ Department of Public Health and Clinical Medicine Umeå University Umeå Sweden; ^7^ Northern Register Centre, Department of Public Health and Clinical Medicine Umeå University Umeå Sweden

**Keywords:** body mass index, interaction, leisure‐time physical activity, obesity‐related cancer

## Abstract

Physical activity (PA) has been associated with a lower risk of some obesity‐related cancers, but the combined association and interaction of PA and body weight on obesity‐related cancer risk is less clear. We examined the association of leisure‐time PA (high/low) and its combination with body mass index (BMI, <25 [low]/≥25 [high] kg/m^2^) on obesity‐related cancer risk in 570 021 individuals, aged 43 years on average at baseline, in five Scandinavian cohorts. We used Cox regression to calculate hazard ratios of obesity‐related cancers (n = 19 074) and assessed multiplicative and additive interactions between PA and BMI on risk. High leisure‐time PA, recorded in 19% of the individuals, was associated with a 7% (95% confidence interval [CI] 4%‐10%) lower risk of any obesity‐related cancer compared to low PA, with similar associations amongst individuals with a low and a high BMI (6% [1%‐11%] and 7% [2%‐11%]). High PA was also associated with decreased risks of renal cell (11% [9%‐31%]) and colon cancer (9% [2%‐16%]). When high PA and low BMI were combined, the relative risk reduction for all obesity‐related cancers was 24% (95% CI 20%‐28%); endometrial cancer, 47% (35%‐57%); renal cell cancer, 39% (27%‐51%); colon cancer, 27% (19%‐35%); multiple myeloma, 23% (2%‐40%) and pancreatic cancer, 21% (4%‐35%), compared to low PA‐high BMI. There were no additive or multiplicative interactions between PA and BMI on risk. The result of our study suggests a reduced risk of obesity‐related cancer by leisure‐time PA in both normal weight and overweight individuals, which further decreased for PA and normal weight combined.

## Graphical abstract

What's new?

Evidence suggests that physical activity lowers risk of certain obesity‐related cancers. Whether physical activity and body weight act together to prevent cancer, however, remains unclear. In this study, the authors explored the impact on obesity‐related cancer risk of associations and interactions between physical activity and body mass index (BMI). While high leisure‐time physical activity and normal weight were individually associated with reduced obesity‐related cancer risk, the greatest risk reduction occurred in the presence of both high physical activity and normal weight. No additive or multiplicative interactions were observed. The findings highlight the importance of physical activity in obesity‐related cancer prevention.

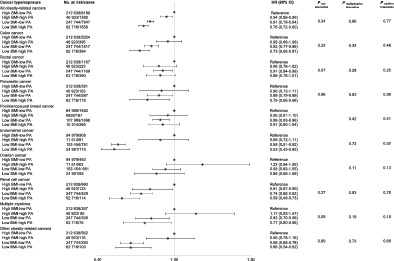

**Abbreviations**
40‐y: Age 40‐programme
BMI: body mass index
CI: confidence interval
HR: hazard ratio
ICD‐7: International Classification of Diseases, 7th edition
MDCS: Malmö Diet and Cancer Study
MET: metabolic equivalent of task
NCS: Norwegian Counties Study
Oslo: Oslo study 1
PA: physical activity
RERI: relative excess risk due to interaction
RR: relative risk
VIP: Västerbotten Intervention Programme

## 1 INTRODUCTION

Cancer is one of the leading causes of death worldwide, and every sixth death in the world is due to cancer, accounting for almost 10 million cancer deaths in 2020.^1^, ^2^ Yet around 40% of cancer cases are estimated to be preventable by avoiding modifiable risk factors including lifestyle‐related factors.^3^, ^4^, ^5^ For physical activity (PA), the association with risk of most individual cancers remains inconclusive.^6^ However, there is strong evidence that PA is associated with a lower risk of colon, postmenopausal breast and endometrial cancer,^6^ and a recent study additionally provided evidence for a potential association with other cancers, in particular kidney cancer and liver cancer.^7^ These same cancers, and another eight to nine cancers, were in the most recent reviews concluded to be obesity‐related with strong evidence.^8^, ^9^

The biological mechanisms through which high PA and normal weight could prevent cancer are likely to be partially shared, for example, through improved immune function, modulated inflammation and enhanced insulin sensitivity.^10^ These shared pathways suggest the potential for an interaction between PA and obesity on cancer risk.^11^ This, however, has only been investigated in one or a few studies each on cancers of the breast, prostate, pancreas, endometrium or colorectum.^12^, ^13^, ^14^, ^15^, ^16^, ^17^, ^18^, ^19^, ^20^, ^21^, ^22^ Some of these studies found a positive multiplicative interaction between low PA and high body mass index (BMI) on risk,^12^, ^14^, ^20^ but most did not.^13^, ^15^, ^16^, ^17^, ^18^, ^19^, ^21^, ^22^ However, interaction on the additive scale is more informative to identify the group at most benefit for intervention, as it reveals the absolute number of cases that could have been prevented in one group vs another.^11^ Additive interaction between PA and BMI on cancer risk has been investigated only in a few studies of various cancers, and these suggested no additive interaction.^12^, ^13^, ^17^

In this pooled cohort study, we investigated the relationship of leisure‐time PA and its combined association and multiplicative and additive interaction with BMI, in relation to the risk of obesity‐related cancers individually and combined.

## 2 METHODS

### 2.1 Study population

We included participants from three Norwegian cohorts: the Oslo study I (Oslo), the Norwegian Counties Study (NCS) and the Age 40 Programme (40‐y), and in two Swedish cohorts: the Västerbotten Intervention Programme (VIP)^23^ and the Malmö Diet and Cancer Study (MDCS). All cohorts are population‐based and include information from health examinations in individuals performed between 1972 and 2014 (see Table 1). The health examination included measurements of height and weight for participants wearing light indoor clothes and no shoes. BMI was calculated by dividing weight in kilogrammes by the square of height in meters (kg/m^2^). Leisure‐time PA was assessed with closed‐ended question/‐s in written questionnaire form. In the Norwegian cohorts, participants were asked to indicate their usual level of leisure‐time PA during the year preceding the survey by selecting one of four categories: (1) reading, watching TV or any other sedentary activity, (2) walking, cycling or other activity for at least 4 hours per week, (3) light sports or heavy gardening for at least 4 hours per week and (4) regular, hard exercise or participating in competitive sports several times a week. In the VIP, participants were asked to select one of five categories to indicate their frequency of exercising in changed outfit with the purpose to increase their fitness level or wellbeing during the last 3 months: (1) never, (2) once in a while, (3) 1 to 2 times a week, (4) 2 to 3 times a week and (5) >3 times a week. In the MDCS, participants were asked to fill in the number of minutes spent per week on 17 leisure‐time PA types separately per four seasons.^24^ A metabolic equivalent of task (MET) value was assigned to each activity. The indicator of PA level is a score calculated as the sum of the number of minutes per week for the four seasons multiplied by MET. Owing to different assessments of PA between the cohorts and, thus, the difficulty to harmonise absolute PA levels between cohorts, in the pooled cohort, we used a similar percentile cut‐point for all cohorts to categorise PA level into low or high. We combined levels 1 to 2 (sedentary to light PA) in the Norwegian cohorts and the VIP as the reference group (low PA), which made up around 80% of the respective population and in the MDCS, we cut the continuous PA variable at the 80th percentile (2962 MET‐min/week). The remaining around 20% of the respective population made up the high PA group (moderate to hard PA; Table S1).

**TABLE 1:** Baseline characteristics of the 570 021 individuals in the study

Characteristics	All	Women	Men
Cohort (year of baseline examination), n (%)			
Total (1972‐2014)	570 021 (100)	284 928 (100)	285 093 (100)
Oslo (1972‐1973)	17 737 (3)	0 (0)	17 737 (6)
NCS (1974‐1988)	90 874 (16)	44 618 (16)	46 256 (16)
40‐y (1985‐1999)	329 943 (58)	171 327 (60)	158 616 (56)
MDCS (1991‐1996)	26 723 (5)	16 056 (6)	10 667 (4)
VIP (1985‐2014)	104 744 (18)	52 927 (18)	51 817 (18)
Age, years			
Mean (SD)	43 (7.5)	44 (7.6)	43 (7.5)
Category, n (%)			
<30	15 514 (3)	7252 (3)	8262 (3)
30‐44	431 219 (76)	218 027 (76)	213 192 (75)
45‐59	87 075 (15)	40 647 (14)	46 428 (16)
≥ 60	36 312 (6)	19 002 (7)	17 211 (6)
Smoking status, n (%)			
Never smoker	229 012 (40)	126 638 (45)	102 374 (36)
Ex‐smoker	128 526 (22)	56 921 (20)	71 605 (25)
Current smoker	209 822 (37)	100 108 (34)	109 714 (38)
Smoking intensity, pack years, n (%)			
<10	75 880 (36)	45 138 (45)	30 742 (28)
10‐19.9	60 240 (29)	30 274 (30)	29 966 (27)
≥20	67 976 (32)	23 487 (24)	44 489 (41)
Pack years missing	5726 (3)	1209 (1)	4517 (4)
Smoking status missing	2661 (1)	1261 (1)	1400 (1)
BMI, kg/m^2^			
Mean (SD)	25 (3.8)	25 (4.1)	26 (3.4)
Category, n (%)			
<25 kg/m^2^ (low BMI)	310 460 (54)	178 435 (63)	132 025 (46)
≥25 kg/m^2^ (high BMI)	259 561 (46)	106 493 (37)	153 068 (54)
Leisure‐time PA, n (%)			
Sedentary to light (low PA)	460 382 (81)	248 433 (87)	211 949 (74)
Moderate to hard (high PA)	109 639 (19)	36 495 (13)	73 144 (26)
Combination of BMI and leisure‐time PA, n (%)			
High BMI‐low PA	212 638 (38)	94 979 (33)	117 659 (41)
High BMI‐high PA	46 923 (8)	11 514 (4)	35 409 (13)
Low BMI‐low PA	247 744 (43)	153 454 (54)	94 290 (33)
Low BMI‐high PA	62 716 (11)	24 981 (9)	37 735 (13)
Follow‐up time, years			
Mean (SD)	20 (8.0)	20 (7.7)	20 (8.3)
Category, n (%)			
<10	57 798 (10)	27 361 (10)	30 437 (11)
10‐19	201 885 (35)	102 378 (36)	99 507 (35)
20‐29	250 214 (44)	128 907 (45)	121 307 (42)
≥30	60 124 (11)	26 282 (9)	33 842 (12)

Abbreviations: 40‐y, Age 40‐programme; BMI, body mass index; MDCS, Malmö Diet and Cancer Study; NCS, Norwegian Counties Study; Oslo, Oslo study 1; PA, physical activity; VIP, Västerbotten Intervention Programme.

### 2.2 Follow‐up

Cancer diagnoses were identified by linking each individual by their unique personal identity number to the respective national Cancer Register in Norway and Sweden. Death and emigration were captured in each national Cause of Death and Population Register, respectively. Follow‐up for these linkages ended on 31 December 2012 for the Norwegian cohorts, on 31 December 2014 for the VIP and on 31 December 2019 for the MDCS. Obesity‐related cancers were defined as those concluded with strong or highly suggestive evidence to be related to obesity in Kyrgio et al.^9^ We also reviewed later Continuous Update Project reports on single cancer forms performed by the World Cancer Research Fund for potential redefinition of obesity‐related cancers, which, however, remained defined as in Kyrgio et al^9^: oesophageal adenocarcinoma (International Classification of Diseases, seventh/tenth edition [ICD‐7/10] code 150/C15, of adenocarcinoma histologic subtype), stomach‐cardia (151.1/C16.0), colon (153/C18), rectum/anus (154/C19‐21), liver/intrahepatic bile ducts (155.0/C22), gallbladder/biliary tract (155.1‐155.3/C23‐24), pancreas (157/C25), postmenopausal breast (170/C50, and attained age ≥60 years), endometrium (172/C54), ovary (175.0/C56), renal cell (180.0, 180.9/C64) and multiple myeloma (203/C90). In our study, we use the term “endometrial cancer” to denote the slightly larger group uterine corpus cancer.

### 2.3 Selection criteria

**FIGURE 1:**
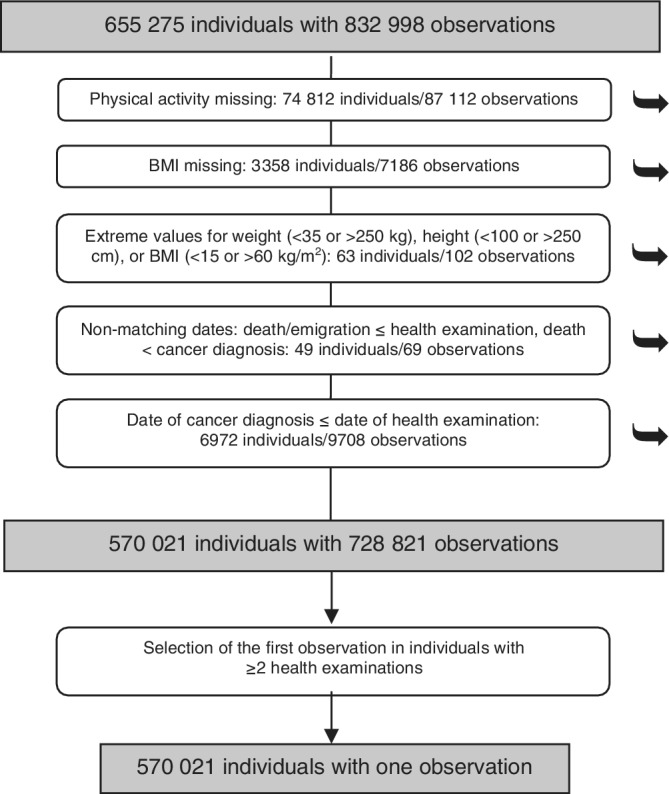
Flowchart of exclusions and selections of individuals and observations in the study. ➥ denotes exclusions

Altogether, the cohorts included 655 275 individuals with 832 998 health examinations (observations). After exclusions due to missing information on leisure‐time PA and BMI, extreme values of height, weight or BMI, mismatching dates and a prevalent cancer (excluding carcinoma in situ and basaliomas), 570 021 individuals with one observation each were retained in the study (Figure 1).

### 2.4 Statistical analysis

Survival analyses were conducted for any obesity‐related cancer and separately by cancer form and by sex if the number of cases was more than 400. Person‐years at risk was calculated from the date of health examination until the diagnosis of any obesity‐related cancer or until censoring due to another cancer, death, emigration or until the end of follow‐up, whichever came first. Cox proportional hazards regression was used to calculate hazard ratios (HRs) with 95% confidence intervals (CIs) of cancer by categories of leisure‐time PA (as previously described), BMI (<25 and ≥25 kg/m^2^) and their combination. Since a small number of individuals and cases were obese (BMI ≥30 kg/m^2^) with moderate to hard PA, we analysed obesity and overweight jointly. We used age as underlying time metric and we adjusted for sex, cohort, date of birth in five categories (before 1931, 1931‐1938, 1939‐1946, 1947‐1954, 1955 and later) and smoking in seven categories (never smoker, ex‐smoker, current smoker by tertile of pack‐years, smokers with pack‐years missing and smoking status missing [1% of individuals]). BMI (continuous) was additionally adjusted for in the analysis of PA and cancer risk as a potential confounder and/or mediator, and the analysis of BMI and cancer risk additionally included adjustment for PA (low/high). Results for BMI and obesity‐related cancer risk have been reported in greater detail before in a population with large overlap with the present study population.^25^, ^26^

Schoenfeld residuals statistics was used to test the proportional hazards assumption of the Cox models. Sex and cohort violated the proportional hazards assumption in some models but including sex or cohort as a stratum in the Cox models did not alter HRs, so they were not retained as stratum in the models.

The relative excess risk due to interaction (RERI) was calculated to investigate additive interactions between PA and BMI in relation to obesity‐related cancer risk. The RERI was based on adjusted HRs representing relative risks (RRs) in the formula: RR11 − RR10 − RR01 + 1, denoting individuals in the low BMI‐high PA group (RR11), low BMI‐low PA group (RR10), high BMI‐high PA group (RR01) and high BMI‐low PA group (1, reference group). CIs were calculated using the delta method.^11^, ^27^ Multiplicative interactions of PA with BMI, cohort and sex, and a three‐way interaction of PA, BMI and sex, were tested by the Wald test of the respective product term in the model. *P*‐values for sex‐interactions were reported for all analyses.

Absolute risks of all incident obesity‐related cancers between 60 and 80 years of age were calculated as described by Gail et al.^28^ For this method, the risks of cancer and of dying from other causes than cancer were derived from the cohort for ages 60 to 70 years and 70 to 80 years, respectively.

All analyses were performed using Stata 16.1, (StataCorp LLC., College Station, Texas).

## 3 RESULTS

The 570 021 individuals (284 928 women, 285 093 men) in the study had a mean baseline age of 43 years (SD = 7.5) (Table 1). Approximately 63% of women and 46% of men had a BMI within the low‐to‐normal range (<25 kg/m^2^), and 13% of women and 26% of men were categorised with moderate to hard PA. After on average 20 years (SD = 8.0) of follow‐up, 19 074 obesity‐related cancer cases (12 075 in women, 6999 in men) had been recorded.

The main associations of leisure‐time PA with cancer risk showed a lower risk of all obesity‐related cancers combined amongst individuals with moderate to hard PA as compared to individuals with sedentary to light PA (HR: 0.93 [95% CI 0.90‐0.96]) (Table 2). This association was similar between individuals with low‐to‐normal weight (HR: 0.94 [95% CI 0.89‐0.99]) and with overweight or obesity (BMI ≥25 kg/m^2^, HR: 0.93 [95% CI 0.88‐0.99]). The inverse association for high PA was also found for colon cancer (HR: 0.91 [95% CI 0.84‐0.98]) and renal cell cancer (HR: 0.79 [95% CI 0.69‐0.91]) (Table 2). Adjusting for BMI minimally attenuated these associations. The associations between PA and obesity‐related cancers risk did not differ between men and women (Table 2) or between cohorts (Table S2). Associations between the original four or five categories of PA in the Norwegian cohorts and in the VIP, and of quartiles of PA in the MDCS, and all obesity‐related cancer risk is shown in Table S3.

**TABLE 2:** Hazard ratio (95% confidence interval) of obesity‐related cancers by level of leisure‐time physical activity

Cancer type	Level of leisure time PA^a^	No. at risk/cases	HR (95% CI)^b^ not BMI‐adjusted	HR (95% CI)^c^ BMI‐adjusted	*P*_sex−interaction_ ^d^
All obesity‐related cancers	All				.67
	Low PA	460 382/16 127	Reference	Reference	
	High PA	109 639/2947	0.93 (0.90‐0.96)	0.94 (0.91‐0.98)	
	Women				
	Low PA	248 433/10 696	Reference	Reference	
	High PA	36 495/1379	0.93 (0.88‐0.98)	0.95 (0.90‐1.01)	
	Men				
	Low PA	211 949/5431	Reference	Reference	
	High PA	73 144/1568	0.92 (0.87‐0.97)	0.94 (0.89‐1.00)	
Colon cancer	All				.67
	Low PA	460 382/3841	Reference	Reference	
	High PA	109 639/779	0.91 (0.84‐0.98)	0.92 (0.85‐1.00)	
	Women				
	Low PA	248 433/1974	Reference	Reference	
	High PA	36 495/249	0.94 (0.82‐1.07)	0.95 (0.84‐1.09)	
	Men				
	Low PA	211 949/1867	Reference	Reference	
	High PA	73 144/530	0.89 (0.80‐0.98)	0.91 (0.83‐1.00)	
Rectal cancer	All				.39
	Low PA	460 382/2336	Reference	Reference	
	High PA	109 639/513	0.93 (0.84‐1.02)	0.93 (0.85‐1.03)	
	Women				
	Low PA	248 433/1045	Reference	Reference	
	High PA	36 495/141	0.99 (0.83‐1.19)	1.01 (0.84‐1.20)	
	Men				
	Low PA	211 949/1291	Reference	Reference	
	High PA	73 144/372	0.91 (0.81‐1.03)	0.92 (0.82‐1.04)	
Pancreatic cancer	All				.92
	Low PA	460 382/1168	Reference	Reference	
	High PA	109 639/221	0.89 (0.77‐1.03)	0.90 (0.78‐1.04)	
	Women				
	Low PA	248 433/560	Reference	Reference	
	High PA	36 495/65	0.89 (0.69‐1.15)	0.90 (0.70‐1.17)	
	Men				
	Low PA	211 949/608	Reference	Reference	
	High PA	73 144/156	0.88 (0.74‐1.06)	0.89 (0.74‐1.06)	
Postmenopausal breast cancer	Women				
	Low PA	171 989/3180	Reference	Reference	
	High PA	22 272/467	0.99 (0.89‐1.09)	0.99 (0.90‐1.09)	
Endometrial cancer	Women				
	Low PA	248 433/1689	Reference	Reference	
	High PA	36 495/201	0.88 (0.76‐1.02)	0.95 (0.82‐1.10)	
Ovarian cancer	Women				
	Low PA	248 433/1144	Reference	Reference	
	High PA	36 495/155	1.02 (0.86‐1.21)	1.03 (0.87‐1.21)	
Renal cell cancer	All				.11
	Low PA	460 382/1189	Reference	Reference	
	High PA	109 639/237	0.79 (0.69‐0.91)	0.82 (0.71‐0.95)	
	Women				
	Low PA	248 433/450	Reference	Reference	
	High PA	36 495/37	0.63 (0.45‐0.89)	0.66 (0.47‐0.93)	
	Men				
	Low PA	211 949/739	Reference	Reference	
	High PA	73 144/200	0.84 (0.72‐0.98)	0.87 (0.74‐1.02)	
Multiple myeloma	All				.05
	Low PA	460 382/666	Reference	Reference	
	High PA	109 639/168	1.03 (0.87‐1.23)	1.05 (0.88‐1.25)	
	Women				
	Low PA	248 433/301	Reference	Reference	
	High PA	36 495/32	0.77 (0.53‐1.11)	0.77 (0.53‐1.11)	
	Men				
	Low PA	211 949/365	Reference	Reference	
	High PA	73 144/136	1.16 (0.95‐1.42)	1.19 (0.97‐1.45)	
Other obesity‐related cancers^e^	All				.07
	Low PA	460 382/955	Reference	Reference	
	High PA	109 639/216	0.95 (0.82‐1.11)	0.99 (0.85‐1.15)	
	Women				
	Low PA	248 433/373	Reference	Reference	
	High PA	36 495/37	0.73 (0.52‐1.03)	0.76 (0.54‐1.07)	
	Men				
	Low PA	211 949/582	Reference	Reference	
	High PA	73 144/179	1.03 (0.87‐1.22)	1.08 (0.91‐1.28)	

Abbreviations: CI, confidence interval; HR, hazard ratio; PA, physical activity.

^a^ Low PA: sedentary to light PA, High PA: moderate to hard PA.

^b^ Hazard ratios from Cox regression models with age as time scale, adjusted for sex, cohort, baseline age, date of birth in five categories (before 1931, 1931‐1938, 1939‐1946, 1947‐1954, 1955 and later) and smoking status and intensity in seven categories.

^c^ Hazard ratios from Cox regression models with age as time scale, adjusted for sex, cohort, baseline age, date of birth in 5 categories (before 1931, 1931‐1938, 1939‐1946, 1947‐1954, 1955 and later), smoking status and intensity in 7 categories and BMI (continuous).

^d^ The *P*‐value for sex‐interaction was based on Wald statistics of the product terms of sex and leisure‐time physical activity in the Cox regression model.

^e^ Other obesity‐related cancers include oesophageal adenocarcinoma, stomach cardia, liver/intrahepatic bile ducts and gallbladder/biliary tract cancer.

Low‐to‐normal weight compared to overweight or obesity was associated with a lower risk of all obesity‐related cancers combined (HR: 0.81 [95% CI 0.79‐0.83]), and of all separate cancers, but only weakly for ovarian cancer for which the confidence interval crossed one (Table S4).

**FIGURE 2:**
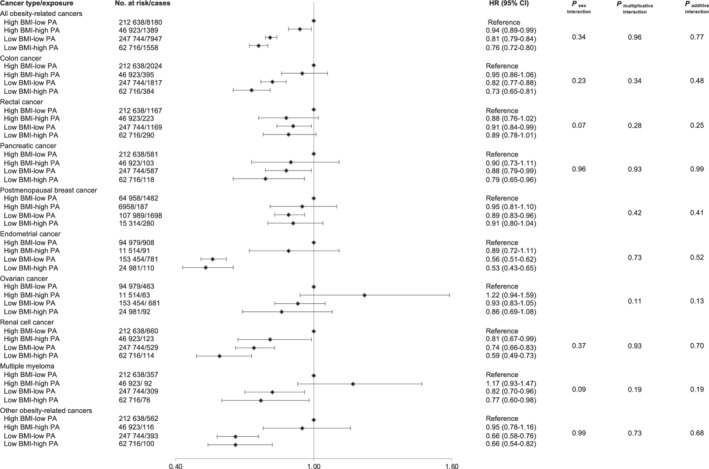
Hazard ratios (95% confidence interval) of obesity‐related cancers in single and combined according to combinations of leisure‐time physical activity and body mass index level. Hazard ratios were calculated by use of Cox regression using age as time scale, adjusted for sex, cohort, baseline age, date of birth and smoking status and intensity. Multiplicative interactions of PA and BMI, and a three‐way interaction of PA, BMI and sex, were tested by the Wald test of the respective product term in the model. Additive interactions of PA and BMI were investigated by calculating the Relative Excess Risk for interaction (RERI) as RR11 − RR10 − RR01 − RR00 + 1, for which the delta method was used to obtain confidence interval. BMI, body mass index; CI, confidence interval; HR, hazard ratio; PA, physical activity. Low PA: sedentary to light exercise, High PA: moderate to hard exercise, Low BMI: <25 kg/m^2^, High BMI: ≥25 kg/m^2^

The associations of a two‐by‐two categorical combination of leisure‐time PA and BMI with the risk of obesity‐related cancers, and *P*‐values from additive and multiplicative interaction tests, are shown in Figure 2. Compared to overweight or obese individuals with low PA, individuals with low‐to‐normal weight and high PA had a lower risk of all obesity‐related cancers (HR: 0.76 [95% CI 0.72‐0.80]), colon cancer (HR: 0.73 [95% CI 0.65‐0.81]), pancreatic cancer (HR: 0.79 [95% CI 0.65‐0.96]), endometrial cancer (HR: 0.53 [95% CI 0.43‐0.65]), renal cell cancer (HR: 0.59 [95% CI 0.49‐0.73]), multiple myeloma (HR: 0.77 [95% CI 0.60‐0.98]) and of the combination of “other” obesity‐related cancers composed of oesophageal adenocarcinoma, stomach‐cardia cancer, liver cancer and gallbladder cancer (HR: 0.66 [95% CI 0.54‐0.82]). BMI appeared to be a stronger driver than PA in their joint association with all obesity‐related cancers, endometrial cancer, multiple myeloma and “other” obesity‐related cancers. There were no additive or multiplicative interactions between PA and BMI for all obesity‐related cancers or specific cancer types. There were no sex‐interactions in the associations; however, sex‐specific results are reported in Figure S1.

In absolute terms, the risk of all incident obesity‐related cancer over a 20‐year period for 60‐year‐old women of low‐to‐normal weight with moderate to hard leisure‐time PA, and with overweight or obesity with sedentary to light leisure‐time PA, was 8.4% and 10.7%, respectively. In men, the corresponding absolute risks were 3.8% and 5.0%, respectively.

## 4 DISCUSSION

In this pooled cohort study, we found that moderate to hard leisure‐time PA compared to sedentary to light leisure‐time PA was associated with a lower risk of all obesity‐related cancers, colon cancer and renal cell cancer. Moderate to hard PA and a BMI in the low‐to‐normal range jointly contributed to a lower risk of obesity‐related cancers and several separate cancers. No additive or multiplicative interaction was found between PA and BMI, and the risk reduction of all obesity‐related cancer by PA was similar amongst low‐to‐normal weight and overweight or obese individuals.

There is strong evidence to support a role of leisure‐time PA in reducing the risk of some cancers, including colon, postmenopausal breast and endometrial cancer.^6^ The evidence for other cancers is less conclusive, but accumulating evidence suggests an association also for other cancers including kidney cancer,^7^, ^29^ which is further supported by the results of our study. Our study did not confirm an association between PA and postmenopausal breast and endometrial cancer risks; however, the results of previous studies have been heterogenous and, for postmenopausal breast cancer, they have shown modest effect sizes.^6^ The heterogenous findings between studies are likely affected by different assessments and cut‐points of PA. For example, the risk of postmenopausal breast cancer has been shown to reduce already with light exercise such as walking,^30^ which could not have been captured in our study due to the higher cut‐point of PA. Moreover, for cancers with a dose‐response effect of PA, such as postmenopausal breast cancer,^6^, ^7^ the effect size will be larger when comparing PA levels in the two ends excluding a middle section than using one single cut‐point such as in our study.

Cancers conclusively or suggestively associated with PA are confirmed to be obesity‐related.^10^ Several biological mechanisms whereby obesity, PA and sedentary behaviour may influence cancer risk have been proposed. For example, obesity and physical inactivity contribute to energy imbalance, which may be linked to cancer through oxidative stress, DNA repair and telomere length.^31^ PA and weight regulation are determinants of energy balance. Maintaining an optimal level of energy balance (caloric expenditure relative to caloric intake) can reduce systemic and adipose tissue inflammation and angiogenesis, alter endogenous hormone metabolism and adipokine levels and improves insulin sensitivity, which are strongly hypothesised biological mechanisms in the development of cancer.^10^, ^32^ These potential joint pathways for PA and obesity on cancer suggest the potential for interaction, that is, a risk or relative risk increase or reduction that only occurs in the presence of both factors.^11^

Few studies have investigated such interaction on cancer. Consistent with our findings, a Danish study showed no interaction between BMI and PA on all cancer incidence.^21^ Similarly, in relation to individual cancer forms, no multiplicative interaction between BMI and PA has been found for cancer of the endometrium,^18^, ^19^ postmenopausal breast,^17^, ^20^ pancreas,^15^, ^16^ and colon,^14^, ^33^ and three studies of breast cancer assessing the additive interaction between BMI and PA on risk found no interaction.^13^, ^17^, ^34^ The modest association between PA and cancer risk in our study may be a reason for why no interaction with BMI was found. Despite this lack of interaction, however, moderate to hard PA and low‐to‐normal weight both contributed to a reduced cancer risk in our study, totalling a 24% relative risk reduction compared to that of inactive, overweight or obese individuals, and an absolute 20‐year risk reduction between 60 and 80 years of age of around 1% to 2%. For all obesity‐related cancers, we observed that BMI was a stronger driver in the joint association with PA on risk than was PA, which was also observed for some separate cancers, especially endometrial cancer. However, the strengths of these associations and individual contribution of PA and BMI in the associations heavily depend on the chosen cut‐points, as previously discussed, and the specific markers used for PA and adiposity.

Our study has some strengths and limitations. Firstly, by use of unique personal identity numbers in Norway and Sweden, we could link individuals to national registers with high completeness and validity.^35^, ^36^, ^37^ Furthermore, the large sample size and long follow‐up, which enabled the investigation also of rarer cancer forms, although for some of these, there were less than 100 cases in certain subgroups limiting statistical power to reach significance. The main limitation of our study, like in the vast majority of observational studies of PA, is the once‐only and self‐reported form of leisure‐time PA, which may include both random and systematic misclassifications^38^, ^39^, ^40^ potentially causing diluted or biased results. Furthermore, the different classifications of PA in each cohort restricted the options for categorisation of PA after pooling. Access to information on MET minutes in all cohorts, instead of in only one of our included cohorts, would have facilitated more options for categorisation and the investigation of a dose‐response relationship. We also lacked information on potentially important confounders for some cancer forms, including dietary intake and, in women, information on reproductive and hormone‐related factors. However, the information on smoking—an important potential confounder for most of the cancers investigated—was virtually complete for both smoking status and pack‐years in current smokers.

In conclusion, our study showed a reduction in obesity‐related, colon and renal cell cancer risk with moderate to hard leisure‐time PA. Higher PA jointly with low‐to‐normal weight was associated with a further reduced risk contributed by both factors, but without evidence of an interaction. Collectively, these findings underscore the importance of PA in the prevention of obesity‐related cancer irrespective of an individual's body weight.

## CONFLICT OF INTEREST

The authors declare no conflicts of interest.

## AUTHOR CONTRIBUTIONS

Ming Sun: Conceptualization, Formal analysis, Funding acquisition, Investigation and Writing‐original draft. Tanja Stocks: Conceptualization, Data curation, Funding acquisition, Investigation and Writing‐review & editing. Tone Bjørge: Data curation, Investigation and Writing‐review & editing. Stanley Teleka, Anders Engeland, Patrik Wennberg, Christel Häggström: Investigation and Writing‐review & editing. The work reported in the article has been performed by the authors, unless clearly specified in the text.

## ETHICS STATEMENT

The study was approved by Ethics Committees in Norway (Regionale komiteer for medisinsk og helsefaglig forskningsetikk, no 2012/2271/REK sør‐øst) and Sweden (EPN Umeå, no 2012‐354‐31M and no 2015‐7‐32M and EPN Lund, no 2014/830). Written informed consent was obtained from participants in the MDCS. Participants in the VIP provided written informed consent for a blood draw, taken at the health examination, to be donated for future research. In Norway, the participants were invited to the health survey and a questionnaire was sent together with the invitation. An attendance to the health examination where the participants delivered their filled in questionnaire has been accepted by the Data Inspectorate as an informed consent, but not a written consent. Written consent was obtained from 1994 onwards.

## Supporting information


**Appendix S1** Supporting Information.

## Data Availability

Data are available from the corresponding author conditional on permission from the involved cohort committees and national registers.

## References

[1] Sung H , Ferlay J , Siegel RL , et al. Global cancer statistics 2020: GLOBOCAN estimates of incidence and mortality worldwide for 36 cancers in 185 countries. CA Cancer J Clin. 2021;71:209‐249. 33538338 10.3322/caac.21660

[2] American Cancer Society . Global Cancer Facts & Figures. 4th ed. Atlanta: American Cancer Society; 2018.

[3] Danaei G , Vander Hoorn S , Lopez AD , Murray CJ , Ezzati M . Causes of cancer in the world: comparative risk assessment of nine behavioural and environmental risk factors. Lancet. 2005;366:1784‐1793. 16298215 10.1016/S0140-6736(05)67725-2

[4] Parkin DM , Boyd L , Walker LC . The fraction of cancer attributable to lifestyle and environmental factors in the UK in 2010. Br J Cancer. 2011;105(Suppl 2):S77‐S81. 22158327 10.1038/bjc.2011.489 PMC3252065

[5] Poirier AE , Ruan Y , Volesky KD , et al. The current and future burden of cancer attributable to modifiable risk factors in Canada: summary of results. Prev Med. 2019;122:140‐147. 31078167 10.1016/j.ypmed.2019.04.007

[6] World Cancer Research Fund/American Institute for Cancer Research . The Third Expert Report, Diet, Nutrition, Physical, Activity and Cancer: a Global Perspective; 2018.

[7] Matthews CE , Moore SC , Arem H , et al. Amount and intensity of leisure‐time physical activity and lower cancer risk. J Clin Oncol. 2020;38:686‐697. 31877085 10.1200/JCO.19.02407 PMC7048166

[8] Lauby‐Secretan B , Scoccianti C , Loomis D , et al. Body fatness and cancer: viewpoint of the IARC working group. N Engl J Med. 2016;375:794‐798. 27557308 10.1056/NEJMsr1606602 PMC6754861

[9] Kyrgiou M , Kalliala I , Markozannes G , et al. Adiposity and cancer at major anatomical sites: umbrella review of the literature. BMJ. 2017;356:j477. 28246088 10.1136/bmj.j477 PMC5421437

[10] Friedenreich CM , Ryder‐Burbidge C , McNeil J . Physical activity, obesity and sedentary behavior in cancer etiology: epidemiologic evidence and biologic mechanisms. Mol Oncol. 2021;15:790‐800. 32741068 10.1002/1878-0261.12772 PMC7931121

[11] Tyler JV , Mirjam JK . A tutorial on interaction. Epidemiol Methods. 2014;3:33‐72.

[12] Grotta A , Bottai M , Adami HO , et al. Physical activity and body mass index as predictors of prostate cancer risk. World J Urol. 2015;33:1495‐1502. 25557943 10.1007/s00345-014-1464-5

[13] Bellocco R , Marrone G , Ye W , et al. A prospective cohort study of the combined effects of physical activity and anthropometric measures on the risk of post‐menopausal breast cancer. Eur J Epidemiol. 2016;31:395‐404. 26130128 10.1007/s10654-015-0064-z

[14] Friedenreich C , Norat T , Steindorf K , et al. Physical activity and risk of colon and rectal cancers: the European prospective investigation into cancer and nutrition. Cancer Epidemiol Biomarkers Prev. 2006;15:2398‐2407. 17164362 10.1158/1055-9965.EPI-06-0595

[15] Jiao L , Berrington de Gonzalez A , Hartge P , et al. Body mass index, effect modifiers, and risk of pancreatic cancer: a pooled study of seven prospective cohorts. Cancer Causes Control. 2010;21:1305‐1314. 20383573 10.1007/s10552-010-9558-x PMC2904431

[16] Stolzenberg‐Solomon RZ , Adams K , Leitzmann M , et al. Adiposity, physical activity, and pancreatic cancer in the National Institutes of Health‐AARP Diet and Health Cohort. Am J Epidemiol. 2008;167:586‐597. 18270373 10.1093/aje/kwm361

[17] McCullough LE , Eng SM , Bradshaw PT , et al. Fat or fit: the joint effects of physical activity, weight gain, and body size on breast cancer risk. Cancer. 2012;118:4860‐4868. 22733561 10.1002/cncr.27433 PMC3448867

[18] Gierach GL , Chang SC , Brinton LA , et al. Physical activity, sedentary behavior, and endometrial cancer risk in the NIH‐AARP Diet and Health Study. Int J Cancer. 2009;124:2139‐2147. 19123463 10.1002/ijc.24059 PMC2845165

[19] Salazar‐Martinez E , Lazcano‐Ponce EC , Lira‐Lira GG , et al. Case‐control study of diabetes, obesity, physical activity and risk of endometrial cancer among Mexican women. Cancer Causes Control. 2000;11:707‐711. 11065007 10.1023/a:1008913619107

[20] Colditz GA , Feskanich D , Chen WY , Hunter DJ , Willett WC . Physical activity and risk of breast cancer in premenopausal women. Br J Cancer. 2003;89:847‐851. 12942116 10.1038/sj.bjc.6601175 PMC2394493

[21] Nunez C , Clausen J , Jensen MT , Holtermann A , Gyntelberg F , Bauman A . Main and interactive effects of physical activity, fitness and body mass in the prevention of cancer from the Copenhagen Male Study. Sci Rep. 2018;8:11780. 30082878 10.1038/s41598-018-30280-5 PMC6078972

[22] Zeegers MP , Dirx MJ , van den Brandt PA . Physical activity and the risk of prostate cancer in The Netherlands cohort study, results after 9.3 years of follow‐up. Cancer Epidemiol Biomarkers Prev. 2005;14:1490‐1495. 15941961 10.1158/1055-9965.EPI-04-0771

[23] Norberg M , Wall S , Boman K , Weinehall L . The Vasterbotten Intervention Programme: background, design and implications. Glob Health Action. 2010;3:4643. 10.3402/gha.v3i0.4643 PMC2844807 20339479

[24] Mutie PM , Drake I , Ericson U , et al. Different domains of self‐reported physical activity and risk of type 2 diabetes in a population‐based Swedish cohort: the Malmo diet and Cancer Study. BMC Public Health. 2020;20:261. 32085709 10.1186/s12889-020-8344-2 PMC7035654

[25] Bjorge T , Haggstrom C , Ghaderi S , et al. BMI and weight changes and risk of obesity‐related cancers: a pooled European cohort study. Int J Epidemiol. 2019;48:1872‐1885. 31566221 10.1093/ije/dyz188

[26] Fritz J , Bjorge T , Nagel G , et al. The triglyceride‐glucose index as a measure of insulin resistance and risk of obesity‐related cancers. Int J Epidemiol. 2020;49:193‐204. 30945727 10.1093/ije/dyz053

[27] Hosmer DW , Lemeshow S . Confidence‐interval estimation of interaction. Epidemiology. 1992;3:452‐456. 1391139 10.1097/00001648-199209000-00012

[28] Gail MH , Brinton LA , Byar DP , et al. Projecting individualized probabilities of developing breast cancer for white females who are being examined annually. J Natl Cancer Inst. 1989;81:1879‐1886. 2593165 10.1093/jnci/81.24.1879

[29] Behrens G , Leitzmann MF . The association between physical activity and renal cancer: systematic review and meta‐analysis. Br J Cancer. 2013;108:798‐811. 23412105 10.1038/bjc.2013.37 PMC3590672

[30] Hildebrand JS , Gapstur SM , Campbell PT , Gaudet MM , Patel AV . Recreational physical activity and leisure‐time sitting in relation to postmenopausal breast cancer risk. Cancer Epidemiol Biomarkers Prev. 2013;22:1906‐1912. 24097200 10.1158/1055-9965.EPI-13-0407

[31] Ulrich CM , Himbert C , Holowatyj AN , Hursting SD . Energy balance and gastrointestinal cancer: risk, interventions, outcomes and mechanisms. Nat Rev Gastroenterol Hepatol. 2018;15:683‐698. 30158569 10.1038/s41575-018-0053-2 PMC6500387

[32] Brown JC , Winters‐Stone K , Lee A , Schmitz KH . Cancer, physical activity, and exercise. Compr Physiol. 2012;2:2775‐2809. 23720265 10.1002/cphy.c120005 PMC4122430

[33] Slattery ML , Potter J , Caan B , et al. Energy balance and colon cancer: beyond physical activity. Cancer Res. 1997;57:75‐80. 8988044

[34] Maliniak ML , Gapstur SM , McCullough LE , et al. Joint associations of physical activity and body mass index with the risk of established excess body fatness‐related cancers among postmenopausal women. Cancer Causes Control. 2021;32:127‐138. 33185805 10.1007/s10552-020-01365-2

[35] Barlow L , Westergren K , Holmberg L , Talback M . The completeness of the Swedish Cancer Register: a sample survey for year 1998. Acta Oncol. 2009;48:27‐33. 18767000 10.1080/02841860802247664

[36] Larsen IK , Smastuen M , Johannesen TB , et al. Data quality at the cancer registry of Norway: an overview of comparability, completeness, validity and timeliness. Eur J Cancer. 2009;45:1218‐1231. 19091545 10.1016/j.ejca.2008.10.037

[37] Laugesen K , Ludvigsson JF , Schmidt M , et al. Nordic Health Registry‐based research: a review of health care systems and key registries. Clin Epidemiol. 2021;13:533‐554. 34321928 10.2147/CLEP.S314959 PMC8302231

[38] Watkinson C , van Sluijs EMF , Sutton S , Hardeman W , Corder K , Griffin SJ . Overestimation of physical activity level is associated with lower BMI: a cross‐sectional analysis. Int J Behav Nutr Phys Act. 2010;7:68. 20854659 10.1186/1479-5868-7-68 PMC2954949

[39] Curtis RG , Olds T , Plotnikoff R , et al. Validity and bias on the online active Australia survey: activity level and participant factors associated with self‐report bias. BMC Med Res Methodol. 2020;20:6. 31924171 10.1186/s12874-020-0896-4 PMC6954551

[40] Slootmaker SM , Schuit AJ , Chinapaw MJ , Seidell JC , van Mechelen W . Disagreement in physical activity assessed by accelerometer and self‐report in subgroups of age, gender, education and weight status. Int J Behav Nutr Phys Act. 2009;6:17. 19320985 10.1186/1479-5868-6-17 PMC2670257

