# Supplementary material for: Interaction of leisure‐time physical activity with body mass index on the risk of obesity‐related cancers: A pooled study
Source: Int J Cancer. 2022 Apr 7;151(6):859–68. doi: 10.1002/ijc.34011 (PMC9546504; doi:10.1002/ijc.34011)
Supplement: Supplementary file 1 — Appendix S1 Supporting Information. [file IJC-151-859-s001.pdf]

Interaction of leisure-time physical activity with body mass index on the risk of obesity-related cancers: a pooled study

Ming Sun, Tone Bjørge, Stanley Teleka, Anders Engeland, Patrik Wennberg, Christel Häggström, Tanja Stocks

Supplementary material contents

Supplementary Tables……………………………………………………………………………………………………… 2

Supplementary Table 1: Cohort-specific definitions and levels for leisure-time physical activity categorization……………………………………………………………………………………………………… 2

Supplementary Table 2: Cohort-specific hazard ratios (95% confidence interval) of all obesity-related cancers by level of leisure-time physical activity in two categories………………………………… 3

Supplementary Table 3: Cohort-specific hazard ratios (95% confidence interval) of all obesity-related cancers by cohort-specific categorical level of leisure-time physical activity…………………… 4

Supplementary Table 4: Hazard ratio (95% confidence interval) of obesity-related cancers by BMI categories……………………………………………………………………………………………………… 5

Supplementary Figures……………………………………………………………………………………………………… 7

Supplementary Figure 1……………………………………………………………………………………………………… 7

Supplementary table 1. Cohort-specific definitions and levels for leisure-time physical activity categorization

Cohort	Norwegian cohorts	VIP	MDCS
**Cohort-specific definition**	During the year preceding the survey, the usual level of PA in leisure time	The frequency of exercising in changed outfit with the purpose to increase their fitness level or wellbeing during the last three months	MET units were multiplied by the sum of the number of minutes per week on 17 leisure-time PA types for the four seasons
**Low PA (reference)** (Sedentary to light PA)	1) Reading, watching TV or any other sedentary activity	1) Never	<2,962 MET-min/week (below 80th percentile of continuous PA variable)
	2) Walking, cycling, or other activity, other for at least 4 hours a week	2) Once in a while<br>3) 1-2 times/week	
**High PA** (Moderate to hard PA)	3) Light sports, heavy gardening (at least 4 hours per week)	4) 2-3 times/week	≥2,962 MET-min/week (above 80th percentile of continuous PA variable)
	4) Regular, hard exercise, or participating in competitive sports several times a week	5) >3 times/week	

Abbreviations: PA, physical activity; VIP, Västerbotten Intervention Programme; MDCS, Malmö Diet and Cancer Study; MET, Metabolic equivalent task.

Supplementary table 2. Cohort-specific hazard ratios (95% confidence interval) of all obesity-related cancers by level of leisure-time physical activity in two categories

**Cohort**	**Level of leisure time PA** [3]	**No. at risk/cases**	**HR (95% CI)** [1] **Not BMI-adjusted**	**HR (95% CI)** [2] **BMI-adjusted**
Norwegian cohorts	Low PA	353,996/10,900	Reference	Reference
	High PA	84,558/2,067	0.92 (0.88-0.97)	0.94 (0.90-0.99)
MDCS	Low PA	21,379/2,386	Reference	Reference
	High PA	5,344/535	0.92 (0.83-1.01)	0.93 (0.84-1.02)
VIP	Low PA	85,007/2,841	Reference	Reference
	High PA	19,737/345	0.91 (0.81-1.02)	0.93 (0.83-1.05)

Abbreviations: HR, hazard ratio; CI, confidence interval; PA, physical activity; VIP, Västerbotten Intervention Programme; MDCS, Malmö Diet and Cancer Study.

1. Hazard ratios from Cox regression models with age as time scale, adjusted for sex, baseline age, date of birth in 5 categories (before 1931, 1931-1938, 1939-1946, 1947-1954, 1955 and later), and smoking status and intensity in 7 categories.
2. Hazard ratios from Cox regression models with age as time scale, adjusted for sex, baseline age, date of birth in 5 categories (before 1931, 1931-1938, 1939-1946, 1947-1954, 1955 and later), smoking status and intensity in 7 categories, and BMI (continuous).
3. Low PA: sedentary to light PA, High PA: moderate to hard PA.

Supplementary table 3. Cohort-specific hazard ratios (95% confidence interval) of all obesity-related cancers by cohort-specific categorical level of leisure-time physical activity

Cohort	Level of leisure time PA	No. at risk/ cases	HR (95% CI) [1] Not BMI-adjusted	HR (95% CI) [2] BMI-adjusted
Norwegian cohorts	Sedentary	86,346/2,643	Reference	Reference
	Light exercise	267,650/8,257	0.98 (0.94-1.03)	1.01 (0.97-1.06)
	Moderate exercise	76,816/1,973	0.94 (0.88-0.99)	0.97 (0.92-1.03)
	Hard exercise	7,742/94	0.60 (0.49-0.74)	0.64 (0.52-0.79)
MDCS	Q1	6,694/764	Reference	Reference
	Q2	6,679/793	1.00 (0.91-1.11)	1.02 (0.92-1.12)
	Q3	6,670/693	0.86 (0.78-0.96)	0.88 (0.80-0.98)
	Q4	6,680/671	0.86 (0.78-0.96)	0.89 (0.80-0.98)
VIP	Never	41,908/1,595	Reference	Reference
	Once in a while	26,079/792	0.88 (0.81-0.96)	0.89 (0.81-0.97)
	1-2 times/week	17,020/454	0.82 (0.73-0.91)	0.84 (0.75-0.92)
	2-3 times/week	13,367/215	0.82 (0.71-0.94)	0.84 (0.73-0.98)
	>3 times/week	6,370/130	0.89 (0.75-1.07)	0.92 (0.77-1.11)

Abbreviations: HR, hazard ratio; CI, confidence interval; PA, physical activity; VIP, Västerbotten Intervention Programme; MDCS, Malmö Diet and Cancer Study.

1. Hazard ratios from Cox regression models with age as time scale, adjusted for sex, baseline age, date of birth in 5 categories (before 1931, 1931-1938, 1939-1946, 1947-1954, 1955 and later), and smoking status and intensity in 7 categories.
2. Hazard ratios from Cox regression models with age as time scale, adjusted for sex, baseline age, date of birth in 5 categories (before 1931, 1931-1938, 1939-1946, 1947-1954, 1955 and later), smoking status and intensity in 7 categories, and BMI (continuous).

Supplementary table 4. Hazard ratio (95% confidence interval) of obesity-related cancers by BMI categories

Cancer type	BMI categories (kg/m^2) [4]	No. at risk/ cases	HR (95% CI) [1]	$P_{sex-interaction}$ [2]
All obesity-related cancers	All			0.84
	High BMI	259,561/9,569	Reference	
	Low BMI	310,460/9,505	0.81 (0.79-0.83)	
	Women			
	High BMI	106,493/5,562	Reference	
	Low BMI	178,435/6,513	0.81 (0.78-0.84)	
	Men			
	High BMI	153,068/4,007	Reference	
	Low BMI	132,025/2,992	0.81 (0.77-0.86)	
Colon cancer	All			0.28
	High BMI	259,561/2,419	Reference	
	Low BMI	310,460/2,201	0.81 (0.76-0.86)	
	Women			
	High BMI	106,493/1,026	Reference	
	Low BMI	178,435/1,197	0.83 (0.77-0.91)	
	Men			
	High BMI	153,068/1,393	Reference	
	Low BMI	132,025/1,004	0.79 (0.72-0.86)	
Rectal cancer	All			0.88
	High BMI	259,561/1,390	Reference	
	Low BMI	310,460/1,459	0.93 (0.86-1.00)	
	Women			
	High BMI	106,493/495	Reference	
	Low BMI	178,435/691	0.91 (0.81-1.02)	
	Men			
	High BMI	153,068/895	Reference	
	Low BMI	132,025/768	0.93 (0.84-1.03)	
Pancreatic cancer	All			0.29
	High BMI	259,561/684	Reference	
	Low BMI	310,460/705	0.88 (0.79-0.98)	
	Women			
	High BMI	106,493/282	Reference	
	Low BMI	178,435/343	0.82 (0.70-0.96)	
	Men			
	High BMI	153,068/402	Reference	
	Low BMI	132,025/362	0.95 (0.82-1.10)	
Postmenopausal breast cancer	Women			
	High BMI	71,916/1,669	Reference	
	Low BMI	122,345/1,978	0.90 (0.84-0.96)	
Endometrial cancer	Women			
	High BMI	106,493/999	Reference	

	Low BMI	178,435/891	0.56 (0.51-0.62)	
Ovarian cancer	Women			
	High BMI	106,493/526	Reference	
	Low BMI	178,435/773	0.90 (0.81-1.01)	
Renal cell cancer	All			0.41
	High BMI	259,561/783	Reference	
	Low BMI	310,460/643	0.74 (0.66-0.82)	
	Women			
	High BMI	106,493/233	Reference	
	Low BMI	178,435/254	0.70 (0.58-0.84)	
	Men			
	High BMI	153,068/550	Reference	
	Low BMI	132,025/389	0.77 (0.67-0.88)	
Multiple myeloma	All			0.003
	High BMI	259,561/449	Reference	
	Low BMI	310,460/385	0.78 (0.68-0.90)	
	Women			
	High BMI	106,493/136	Reference	
	Low BMI	178,435/197	1.01 (0.81-1.26)	
	Men			
	High BMI	153,068/313	Reference	
	Low BMI	132,025/188	0.66 (0.55-0.79)	
Other obesity-related cancers [3]	All			0.77
	High BMI	259,561/678	Reference	
	Low BMI	310,460/493	0.67 (0.59-0.75)	
	Women			
	High BMI	106,493/205	Reference	
	Low BMI	178,435/205	0.69 (0.57-0.85)	
	Men			
	High BMI	153,068/473	Reference	
	Low BMI	132,025/288	0.66 (0.57-0.77)	

Abbreviations: BMI, body mass index; HR, hazard ratio; CI, confidence interval.

1 Hazard ratios from Cox regression models with age as time scale, adjusted for sex, cohort, baseline age, date of birth in 5 categories (before 1931, 1931-1938, 1939-1946, 1947-1954, 1955 and later), smoking status and intensity in 7 categories, and level of leisure-time physical activity.

2 The *P*-value for sex-interaction was based on Wald statistics of the product terms of sex and leisure-time physical activity in the Cox regression model.

3 Other obesity-related cancers include oesophageal adenocarcinoma, stomach cardia, liver/intrahepatic bile ducts, and gallbladder/biliary tract cancer.

4 Low BMI: <25 kg/m^2, High BMI: ≥25 kg/m^2.

Cancer type/exposure	No. at risk/cases	HR (95% CI)	*P* multiplicative interaction	*P* additive interaction
All obesity-related cancers				
High BMI-low PA	94,979/5,004	Reference	0.57	0.44
High BMI-high PA	11,514/558	0.93 (0.85-1.01)		
Low BMI-low PA	153,454/5,692	0.81 (0.78-0.84)		
Low BMI-high PA	24,981/821	0.77 (0.72-0.83)		
Colon cancer				
High BMI-low PA	94,979/927	Reference	0.68	0.63
High BMI-high PA	11,514/99	0.92 (0.75-1.13)		
Low BMI-low PA	153,454/1,047	0.83 (0.76-0.91)		
Low BMI-high PA	24,981/150	0.81 (0.68-0.96)		
Rectal cancer				
High BMI-low PA	94,979/454	Reference	0.05	0.03
High BMI-high PA	11,514/41	0.78 (0.56-1.07)		
Low BMI-low PA	153,454/591	0.87 (0.77-0.99)		
Low BMI-high PA	24,981/100	1.00 (0.80-1.24)		
Pancreatic cancer				
High BMI-low PA	94,979/255	Reference	0.92	0.98
High BMI-high PA	11,514/27	0.92 (0.61-1.36)		
Low BMI-low PA	153,454/305	0.82 (0.69-0.98)		
Low BMI-high PA	24,981/38	0.73 (0.52-1.03)		
Renal cell cancer				
High BMI-low PA	94,979/215	Reference	0.41	0.77
High BMI-high PA	11,514/18	0.76 (0.47-1.23)		
Low BMI-low PA	153,454/235	0.71 (0.59-0.86)		
Low BMI-high PA	24,981/19	0.41 (0.25-0.65)		
Multiple myeloma				
High BMI-low PA	94,979/120	Reference	0.09	0.12
High BMI-high PA	11,514/16	1.11 (0.66-1.88)		
Low BMI-low PA	153,454/181	1.07 (0.85-1.36)		
Low BMI-high PA	24,981/16	0.63 (0.37-1.06)		
Other obesity-related cancers				
High BMI-low PA	94,979/189	Reference	0.82	0.58
High BMI-high PA	11,514/16	0.72 (0.43-1.20)		
Low BMI-low PA	153,454/184	0.69 (0.56-0.85)		
Low BMI-high PA	24,981/21	0.54 (0.34-0.84)		

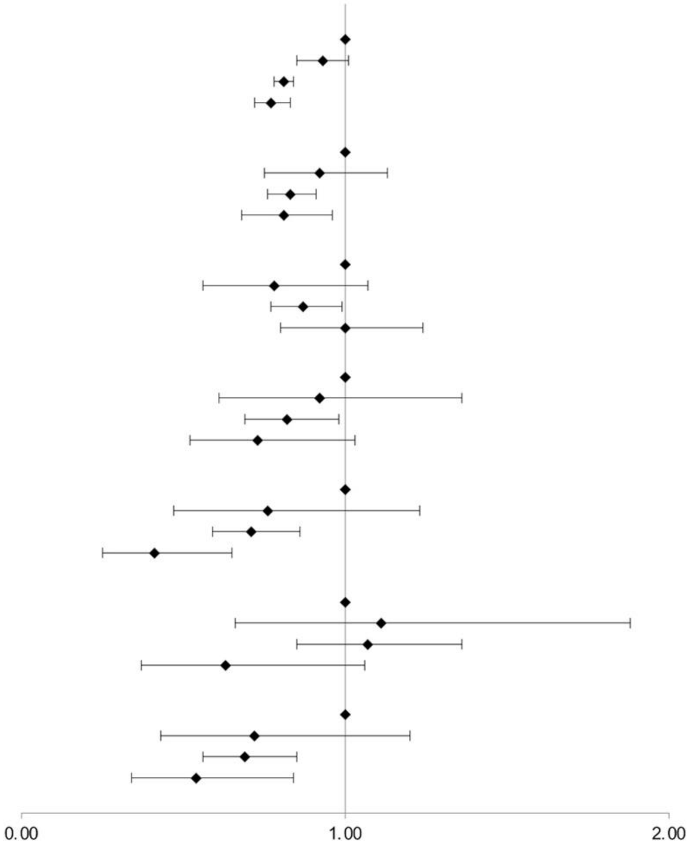

(A) Women

Cancer type/exposure	No. at risk/cases	HR (95% CI)	*P* multiplicative interaction	*P* additive interaction
All obesity-related cancers				
High BMI-low PA	117,659/3,176	Reference		
High BMI-high PA	35,409/831	0.95 (0.88-1.02)	0.55	0.74
Low BMI-low PA	94,290/2,255	0.82 (0.77-0.86)		
Low BMI-high PA	37,735/737	0.75 (0.69-0.81)		
Colon cancer				
High BMI-low PA	117,659/1,097	Reference		
High BMI-high PA	35,409/296	0.96 (0.84-1.09)	0.16	0.26
Low BMI-low PA	94,290/770	0.81 (0.74-0.89)		
Low BMI-high PA	37,735/234	0.68 (0.59-0.78)		
Rectal cancer				
High BMI-low PA	117,659/713	Reference		
High BMI-high PA	35,409/182	0.92 (0.78-1.09)	0.94	0.98
Low BMI-low PA	94,290/578	0.93 (0.83-1.04)		
Low BMI-high PA	37,735/190	0.85 (0.72-1.00)		
Pancreatic cancer				
High BMI-low PA	117,659/326	Reference		
High BMI-high PA	35,409/76	0.90 (0.70-1.16)	0.85	0.87
Low BMI-low PA	94,290/282	0.96 (0.81-1.12)		
Low BMI-high PA	37,735/80	0.83 (0.65-1.07)		
Renal cell cancer				
High BMI-low PA	117,659/ 445	Reference		
High BMI-high PA	35,409/105	0.83 (0.67-1.03)	0.74	0.54
Low BMI-low PA	94,290/294	0.76 (0.65-0.88)		
Low BMI-high PA	37,735/1,995	0.67 (0.53-0.83)		
Multiple myeloma				
High BMI-low PA	117,659/ 237	Reference		
High BMI-high PA	35,409/76	1.15 (0.89-1.49)	0.66	0.93
Low BMI-low PA	94,290/128	0.64 (0.52-1.80)		
Low BMI-high PA	37,735/60	0.81 (0.61-1.08)		
Other obesity-related cancers				
High BMI-low PA	117,659/373	Reference		
High BMI-high PA	35,409/100	1.01 (0.81-1.26)	0.55	0.64
Low BMI-low PA	94,290/209	0.64 (0.54-0.77)		
Low BMI-high PA	37,735/79	0.72 (0.56-0.92)		

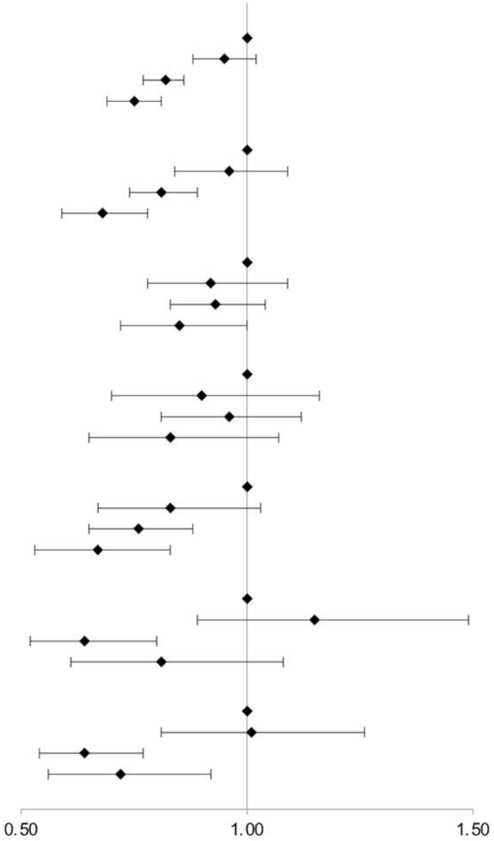

(B) Men

Supplementary figure 1. Hazard ratios (95% confidence interval) of obesity-related cancers in single and combined according to combinations of leisure-time physical activity and body mass index level separately for Women (A) and Men (B). Hazard ratios were calculated by use of Cox regression using age as time scale, adjusted for sex, cohort, baseline age, date of birth, and smoking status and intensity. Multiplicative interactions of PA and BMI were tested by the Wald test of the respective product term in the model. Additive interactions of PA and BMI were investigated by calculating the Relative Excess Risk for interaction (RERI) as RR11-RR10-RR01-RR00+1, for which the delta method was used to obtain confidence interval. PA, physical activity; BMI, body mass index; HR, hazard ratio; CI, confidence interval. Low PA: sedentary to light PA, High PA: moderate to hard PA, Low BMI: <25 kg/m^2, High BMI: ≥25 kg/m^2.
